# Role of circulating tumor cell spheroids in drug resistance

**DOI:** 10.20517/cdr.2019.47

**Published:** 2019-09-19

**Authors:** Gerhard Hamilton, Barbara Rath

**Affiliations:** Department of Surgery, Medical University of Vienna, Vienna A-1090, Austria.

**Keywords:** Cancer, spheroid, circulating tumor cells, small cell lung cancer, non-small-cell lung cancer, transformation, dormancy

## Abstract

Cancer cell spheroids are used for drug screening as these three-dimensional (3D) assemblies recapitulate tumors more realistic than the widely employed 2D *in vitro* cultures. Limited drug diffusion and gradients of oxygen and nutrients in spheroids represent avascular tumor regions containing quiescent and hypoxic tumor cells with high drug resistance. Circulating tumor cells (CTCs) effect metastatic spread and are present in high numbers in malignant diseases such as small-cell lung cancer (SCLC) and in other cancer patients with high tumor load. CTCs are heterogeneous and only a small fraction of these cells survive in the circulation and cause distal lesions. CTCs may circulate as single cells but small CTC clusters or CTC spheroids have been detected in cancer patients and demonstrated to possess increased metastatic potential. At our lab we have obtained 9 permanent SCLC CTC cell lines (BHGc7, BHGc10, BHGc16, BHGc26, BHGc27, BHGc50, BHGc59, BHGc71, and UHGc5) of distinct patients exhibiting similar characteristics and spontaneous formation of large spheroids, termed tumorospheres. These aggregates were shown to exhibit high drug resistance compared to the corresponding single cell suspensions. The increased metastatic capability of small circulating tumor clusters/spheroids may be explained by their role as putative precursors of tumorospheres eventually trapped in capillaries. Limited drug penetration and the presence of hypoxic/quiescent cells can readily account for the global drug resistance of advanced SCLC which has resulted in clinical failure of a wide range of chemotherapeutics and low survival. Furthermore, we have detected such tumorospheres in non-small-cell lung cancer (NSCLC) patients progressing under EGFR-directed tyrosine kinase inhibitor therapy which had undergone NSCLC-SCLC transformation.

## Introduction

Cancer still remains as one of the diseases with highest mortality affecting the worldwide population^[1]^. Since many patients present with metastatic disease or the tumors progress to a disseminated stage despite treatment, cytotoxic or targeted chemotherapy is essential for further care. However, drug resistance limits the efficacy of chemotherapeutics in cancer and, eventually, results in treatment failures and a dismal prognosis. Mechanisms of chemoresistance work at the single cell level but also as a result of the formation of multicellular clusters and at the tumor tissue/tumor microenvironment interphase^[2]^. For individual cells, reduced drug uptake, increased drug efflux, drug inactivation as well as target modifications and impaired execution of cell death are the most important mechanisms whereas at the tumor level limited drug diffusion, high interstitial pressure, extracellular acidosis, cell contact-mediated mechanisms and irregular vessel supply contribute to refractoriness to treatment^[3]^. Avascular tumors or metastases often display a gradient in oxygen levels leading to a proliferative zone and a hypoxic core with quiescent cells that are more resistant to chemotherapy, immunotherapy and irradiation^[4]^.

For successful preclinical drug development, the compounds need to be tested using model systems recapitulating the features of native tumors accurately. Historically, simple two-dimensional (2D) cultures of permanent cancer cell lines and animal models were employed which resulted in a high attrition rate in subsequent clinical trials due to a lack of predictive power^[5]^. Up to 95% of anticancer drugs in clinical trials failed due to inefficacy and toxicity despite showing efficacy during preclinical testing^[6]^. To achieve better predictive tests, three-dimensional (3D) cell culture systems were developed because they promised to resemble the *in vivo* tissue environment more precisely than 2D systems^[7,8]^. Of the various 3D tumor models under investigation, the most common are the spheroids which can be prepared by a range of techniques relying simply on the prevention of cancer cell attachment to substrates^[9]^. Spheroids are scaffold-free globular self-assembled aggregates of cancer cells which exhibit an intermediate complexity between 2D *in vitro* cell cultures and *in vivo* solid tumors^[10]^. Advanced 3D models include cocultures of tumor cells with cancer-associated fibroblasts (CAFs) and immune cells, as well as tumor ECM components^[9]^. Comparison of spheroids with conventional 2D cell monolayers has revealed marked differences in the response to anticancer drugs^[11]^. In dependence of their size, spheroids display higher chemoresistance due to contact-mediated resistance, exclusion of drugs and their content of quiescent and hypoxic cells as a result of gradients of nutrients and oxygen^[10]^. In large spheroids, a necrotic core is present when the innermost cells are located beyond the diffusion limit of oxygen approximately 200 μm from the surface of the cellular aggregate^[12-14]^. This mimics solid tumors because delivery of oxygen to the cells present in the inner regions of the tumor mass is frequently reduced due to the increased oxygen diffusion distances^[15,16]^. In summary, on the one hand, spheroids represent an important tool for *in vitro* drug screening and on the other hand, specific multicellular assemblies of cancer cells possibly present in patients may be an important mechanism of resistance to chemotherapy.

## Tumor spheroids as an *in vitro* drug screening tool

**Figure 1:**
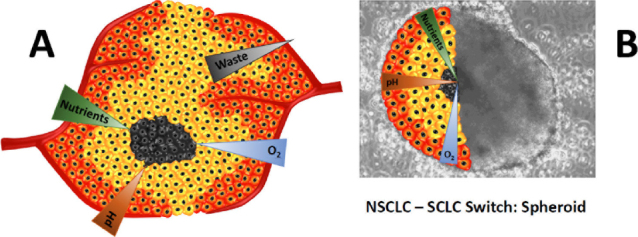
A: Depicts a poorly vascularized tumor region showing gradients of oxygen, nutrients and waste; B: shows an overlay of a large tumorosphere of a patient which has transformed from NSCLC under Osimertinib treatment to SCLC. The corresponding gradients are shown for the tumorosphere. NSCLC: non-small-cell lung cancer; SCLC: small-cell lung cancer

Tumor spheroids seem to provide a better representation of real malignant tissues and are used in drug screening in different forms, depending on the method of preparation. In principle, spheroids can be generated using the hanging drop technique, suspension/spinner flasks, ultra-low attachment plates, micro-patterned plates or microfluidics devices, to mention a few procedures^[17]^. Most cell lines form spheroids under the appropriate non-adherent conditions but properties of these structures vary widely in respect to size, surface density, gradients of nutrients and oxygen as well as homogeneity of the preparations. The transport of oxygen, nutrients and metabolic wastes through the layers of the spheroid is decelerated such creating specific gradients which mimic a physical barrier [Figure 1A]. Drug transport into cells is tightly controlled by hurdles including the plasma membrane, transport proteins, cellular efflux pumps, cell adhesion molecules and gap junctions^[18]^. The ability to cross these barriers *in vivo* determines drug absorption, distribution, metabolism, excretion, and toxicity (ADME-Tox).

Large spheroids (~ 400-500 μm diameter) display a layered cell distribution analogous to that observed in solid tumors^[19]^. Accordingly, well-nourished cells in the outer layer exhibit high proliferation whereas the middle layer is characterized by quiescent cells and the depleted core may contain necrotic cells and acellular regions. The presence of diffusive gradients across the spheroids account for the impaired cytotoxicity of various anticancer drugs and of irradiation. Cells in the hypoxic region are resistant to drugs or irradiation which kill cells through reactive oxygen species, while quiescent cells are all less sensitive to drugs requiring active cell cycling. Furthermore, spheroids possess a network of structural and adhesive ECM proteins which regulate response to growth factors, cell proliferation and differentiation^[20]^. In addition, spheroids closely mimic the physical barriers found in real solid tumors, which obstruct the free penetration of drugs through the whole mass. However, a limiting fact of spheroids is the lack of relevant features of real tumors such as the supporting vasculature, effector cells of the immune system and the fluid dynamics. Furthermore, ECM components expressed in spheroids are of different cellular origin compared to the range of proteins supplied by CAFs *in vivo*.

The response of spheroids to cytotoxic drugs can be assessed by variation of morphometric parameters such as their diameter, volume and circularity from bright field microscopy by image analysis. Other methods rely on measurements of membrane integrity (i.e., LDH assay), intracellular mitochondrial activity (e.g., formazan dyes MTT, WTS-1, resazurin dye AlamarBlue) or ATP content. Alternatively, the effect of drugs on spheroids can be analyzed using immunofluorescence staining, expression of fluorescent proteins and fluorescent probes for checking the state of the constituting cells^[21]^. However, assays such as formazan or resazurin reduction assays may not be efficient with spheroids because of the incomplete probe penetration.

Distinct cancer cell lines and techniques for generating spheroids produce a wide variety of such multicellular aggregates with an extreme range of molecular and physical properties. Exclusion of probes or drugs by spheroids are an important test to confirm the presence of a tight physical surface barrier and to prove the actual representation of an avascular tumor region. Still, the majority of spheroids for drug screening uses permanent cell lines which has been adapted to tissue culture conditions for prolonged times and have lost the lower proliferation rates and various characteristics of primary tumor cells. Most of these spheroids are loose assembly of cancer cells and provide no significant barrier to drug penetration and cytotoxicity^[22]^. The production of large spheroids is difficult since cells tend to adopt irregular shapes and are not any longer suitable for screen drugs in a reproducible manner. Inclusion of CAFs and immune cells in scaffold-embedded structures are technically demanding and significantly increase costs and effort for screening. Additionally, the proof that advanced 3D models show superior predictive power for the selection of promising drugs is largely lacking. The spheroid models are relatively simple with respect to clinical tumors and, therefore, the reliability of their drug-response prediction capacity should not be overestimated^[23]^.

## Ovarian and lung cancer spheroids

Spheroids can be easily generated *in vitro* by different methods but such aggregates are found similarly *in vivo* under special conditions, namely in ascitic fluid and pleural effusions in ovarian and lung cancer among others, respectively. Epithelial ovarian cancer (EOC) is the most lethal gynecological cancer, with a 5-year survival rate below 30% because the current chemotherapeutic regimes for EOC are unable to achieve sustained remission^[24]^. EOC exhibits local invasion and this peritoneal carcinomatosis is present in a third of patients at diagnosis and in almost all patients at recurrence. For EOC, *in vitro* cultured spheroids are capable of tumorigenesis *in vivo* and display a reduced response to chemotherapeutic drugs when compared to monolayers^[25]^. Therefore, spheroid formation promotes chemoresistance and represents a key component of platinum- and taxane-sensitive recurrence in EOC. Spheroids of EOC arise from collective detachment, rather than aggregation and are more resistant to anoikis than single cells^[26]^. Subsequently, spheroids can reattach to the mesothelium and invade the ECM during dissemination. In patients, a wide range of sizes of spheroids were observed comprising clusters ranging from less than 20 cells to over 100 cells with approximately 40% of cells positive for the proliferation marker Ki67^[27]^. The EOC spheroid generates a metabolite gradient that inhibits access of chemotherapy agents to the internal cells and the resulting slowly cycling and/or quiescent cellular states are resistant to therapies involving platinum drugs and taxanes that specifically hit proliferating cells^[28]^.

Lung cancer is the most common cause of cancer deaths worldwide. The two broad histological subtypes of lung cancer are small-cell lung cancer (SCLC), which is the cause of 15% of cases, and non-small-cell lung cancer (NSCLC), which accounts for 85% of cases and includes adenocarcinoma, squamous-cell carcinoma, and large-cell carcinoma^[29]^. In advanced stage, malignant pleural effusions (MPEs) are frequently present and constitute an excellent source to culture a wide variety of cancer cells, in particular NSCLC classified as adenocarcinomas^[30,31]^. In contrast to pleural cells, establishment of cancer cell lines from solid tumor tissue is frequently inefficient, because of loss of growth after a few passages. MPE is caused by a combination of processes such as inflammation, enhanced angiogenesis and vascular leakage. Withdrawal of ascites or pleural effusions is often performed to relieve symptoms and is less invasive than tumor biopsies. These patients have a poor prognosis with median survival times ranging from 2.5 months in lung cancer to approximately 17 months for mesothelioma^[32]^. Such MPE gave rise to *in vitro* cultures both in adherent and/or in spheroid conditions in almost all cases. These cultures form large irregular spheroids in non-adherent regular medium or smaller and very compact spheres in serum-free sphere medium and, furthermore, exhibit efficient tumor engraftment in immunocompromised mice.

In general, pleural lung cancer spheroids are less than 200 µm in diameter and, in our experience, these aggregates tend to disperse and to grow as monolayers^[22,33]^. Generation of cultures from these tumor cells has a high success rate, thereby amenable for sensitivity tests and selection of therapy^[34]^. For example, single multicellular spheroids from malignant pleural mesothelioma were trapped and tested for chemotherapeutic drug response on a microfluidic platform^[35]^. Adenocarcinoma MPEs of NSCLC resulted in *in vitro* cultures both in adherent and/or in spheroid conditions but the ability of such preparations to induce xenografts appeared to correlate with the production of large spheres and not with a particular expression of known surface markers^[36]^. In conclusion, spheroids are present in malignant peritoneal fluid and pleural effusions but these aggregates tend to exhibit smaller sizes without the hypoxic and necrotic regions observed in larger 3D structures. In these cases, chemoresistance seem to be produced by cell-cell contact, altered gene expression and concurrent resistance to anoikis-induced cell death. Cell-cell interactions in spheroids can control cell signaling, survival, proliferation, and drug sensitivity, in particular under control of E-cadherins^[37]^.

## Circulating tumor cell clusters

The major cause of cancer-associated mortality is tumor metastasis which is mediated by circulating tumor cells (CTCs) detached from the primary lesion. CTCs have been detected in variable numbers in almost all tumors with highest numbers in SCLC and inflammatory breast cancer (IBC)^[38]^. The number of CTCs has predictive power and their variation in response to therapy indicates the efficacy of treatment. Only a minor fraction of CTCs survives in the circulation, extravasates and is able to establish metastases. Many studies have monitored CTCs in patients’ blood and the investigations showed that, in contrast to single cells, CTC clusters have a high metastastic potential^[39,40]^. These structures have been described as circulating tumor microemboli, circulating micrometastases or circulating tumor aggregates and are defined as groups of tumor cells that travel together in the bloodstream^[41]^. Thus, CTCs can exist as single cells or clusters, ranging from two cells to dozens of cells but this number of cells is too low to constitute spheroids with hypoxic cores. Several mechanisms are supposed to account for the higher metastatic potential of CTC clusters and a correspondingly poor prognosis such as an increased lodging in small blood vessels in various organs, increased resistance to cell death after homing in distal organs and acquisition of stem cell signatures within the cell assemblies^[42,43]^.

CTC clusters are expected to have a very short life span in the circulation due to a faster removal by filtration in the circulation compared to single cells^[44]^. Studies showed that the formation of metastases partially depended on the size and concentration of CTC clusters^[45,46]^. In patients with metastatic breast and cervical carcinomas, tumor cell emboli entrapped in vessels in were proved in the microvasculature of the lungs^[47]^. The size distribution of vessels was an important determinant of the distribution and survival of CTC clusters^[45]^. However, a delayed aggregation of CTCs at inflammatory sites or after entrapment in small vessels could not be ruled out^[48]^. In SCLC, the presence of CTC cluster was reported to be associated with shorter progression-free and overall survival^[41]^. Although the metastatic process cannot be monitored in patients, CTC clusters may be the precursors of large spheroids in small vessels in advance of extravasation and growth of secondary lesions. Cardiac glycosides were found to disintegrate CTC cluster and were proposed as antimetastatic agents^[42]^. However, the actual tumor dissemination via CTCs may occur at a time when the initial tumor is of small size and has not been detected clinically thus making a late CTC-directed intervention a futile effort^[49]^.

## Small cell lung cancer spheroids

SCLC is a highly aggressive malignancy with a dismal prognosis found mostly associated with heavy tobacco consumption^[50]^. This tumor is disseminated in most cases at first presentation and while exhibiting high initial response rates to first-line platinum-based chemotherapy the disease relapses invariably within approximately 1 year and second-line treatments with either topotecan or epirubicin-based regimens yield low response rates of short duration. Except a minor extension of survival by novel checkpoint immunotherapy, no progress has been made for the last decades with no new drugs approved^[51,52]^. There is no lack of clinical trials - the NIH clinical trial registry counts over 800 investigations comprising new diagnostic methods, novel drugs, new combinations of drugs and alternating treatment regimens. The therapeutics tested clinically target a host of pathways and effectors ranging from novel platinum compounds, camptothecines, cell cycle inhibitors, PARP inhibitors, modulators of apoptosis and many others. So far, no definite mechanisms of resistance were reported for advanced SCLC which could have been exploited for novel therapeutic strategies. Instead, numerous therapeutics with anticancer activities shown in other malignancies have been administered to SCLC patients and largely demonstrated to be more or less tolerated but to fail to yield significant responses. Genomics has demonstrated inactivation of tumor suppressor proteins p53 and RB1 with broad alterations in the regulation of transcription and putative drugable modifications in small subpopulations of patients.

The rapid dissemination of SCLC is associated with excessive numbers of CTCs in many patients exceeding the numbers of this cell type detectable in colorectal and breast cancers by orders of magnitude. EpCAM-positive SCLC CTCs have been counted using the CellSearch system and other techniques and correlated with prognosis and response to therapy in a range of studies. Furthermore, enriched SCLC CTC fractions were demonstrated to be tumorigenic in immunocompromised mice. In general, CTCs are highly heterogeneous and only a small fraction is competent in inducing metastases. At our institution we were able to initiate 9 permanent SCLC CTC lines (BHGc7, BHGc10, BHGc16, BHGc26, BHGc27, BHGc50, BHGc59, BHGc71, and UHGc5) from SCLC patients with a higher tumor load. This panel of SCLC CTC cell lines exhibit the typical SCLC markers, such as chromogranin A, enolase-2, synaptophysin and specific p53 mutations. Upon growth in tissue culture, all CTCs derived from the different patients developed spontaneously large multicellular aggregates up to 1 mm, termed tumorospheres according to a nomenclature proposed by Weiswald *et al*.^[53]^. In contrast to clusters and spheroids found in pleura and ascites, tumorospheres consist of 10^4^-10^6^ tumor cells. Proliferation marker Ki67 or PCNA as well as carbonic anhydrase IX (CAIX) staining of tumorosphere sections revealed a rim of proliferating cells and a layer of quiescent and hypoxic cells surrounding a necrotic core region, respectively. Accordingly, comparison of the chemosensitivity of CTC single cell suspensions with tumorospheres demonstrated markedly increased resistance of the clusters against chemotherapeutics commonly used for treatment of SCLC^[54]^. Therefore, global chemoresistance of relapsing SCLC seems to rely on formation of large tumorospheres which reveal limited accessibility for drugs, lower growth fraction and a hypoxic inner microenvironment. The same spheroid-forming ability of SCLC CTCs derived from blood samples of 9 different patients and their similarity of chemosensitivities and molecular markers point to an important role in relapsed SCLC patients^[54-56]^. Actually, all SCLC CTC lines were established from patients with extended metastatic disease and a high tumor load after 2nd line therapy with poor prognosis, such suggesting an important role of these spheroids in chemoresistance and fatal tumor progression. The CTC tumorospheres show a rather uniform size distribution, depending on the specific CTC line, and may be expanded to large spheroid numbers which are suitable for advanced drug screening. SCLC seems to represent a unique tumor model to study the association of CTCs, metastasis and drug resistance. Of course, tumorospheres cannot exist in the circulation for long but SCLC CTCs or SCLC CTC clusters may be released by primary tumors after first-line chemotherapy or may grow to these large spheroids from smaller aggregates after entrapment in the microvasculature as observed under tissue culture conditions.

SCLC was designated as “graveyard of drug development” since a host of drugs with different targets failed clinically and over 800 studies registered by the US clinical trials site brought little progress in therapeutic modalities with the single exception of a minor prolongation of survival in response to immunotherapy^[52]^. However, SCLC seems to constitute an example of the failure of drug screening that is confined simply to 2D cell line models and loose aggregates of cancer cell lines in suspension. Although SCLC *in vivo* exhibits aggressive proliferation which results in avascular regions and necrotic areas, drug screening was invariably performed without attempts to use 3D models. Tumorospheres with quiescent cells and hypoxic/necrotic regions are physically resistant to a host of drugs without requiring special molecular mechanisms of drug resistance. In conclusion, CTC tumorospheres represent natural grown and large spheroids which are expected to be involved in tumor dissemination and drug resistance in SCLC and provide a suitable source for 3D testing of drugs. To overcome this type of resistance means to disintegrate spheroids *in vivo* would be required.

## Histological transformation of NSCLC to SCLC

In 3%-15% of patients with NSCLC tumors, transformation to SCLC can occur, preferentially during progression under tyrosine kinase inhibitor (TKI) or rarely under immune checkpoint therapy^[57-60]^. Concurrent development of adenocarcinoma and SCLC was noted in EGFR-mutant tumors before treatment with an EGFR inhibitor^[61]^. Approximately after one year of EGFR-directed TKI therapy, resistance develops comprising several distinct mechanisms including the most common EGFR T790M mutation in 50%-60% of tumor samples^[62]^. Resistance mechanisms that bypass EGFR signaling, such as MET and HER2 amplification account for another 15%-20% of resistance to EGFR inhibitors and a less common mechanism is the histological transformation of EGFR-mutant adenocarcinoma to SCLC^[63,64]^. This process was first described in 2006 in a patient originally diagnosed with adenocarcinoma revealing SCLC with the original EGFR exon 19 deletion mutation in a repeat biopsy^[65]^. Since then, several other case series have been reported and these SCLCs were identified by morphology and positive immunohistochemical staining for synaptophysin, chromogranin, or NCAM^[65-67]^.

Switch from NSCLC to SCLC is a mechanism of resistance in EGFR-mutant tumors which retain the specific mutation^[68]^. This suggests that these SCLCs were not independent de-novo cancers, but a transformed phenotype^[63]^. SCLC transformation occurs earlier in EGFR-mutant patients but both groups were responsive to platinum/etoposide regimens with approximately 40% response rate. Transformation to SCLC suggests that both adenocarcinoma and SCLC arise from a common cell type^[63,64,69]^. The initial response to chemotherapy is much greater for patients with advanced SCLC than patients with metastatic NSCLC which suggests inherent differences in tumor biology. Two large case series have investigated the frequency of tumors with combined SCLC and NSCLC histology and reported 2%-10% of such tumors^[70,71]^. Although EGFR mutations are identified in SCLC, these patients have had mixed responses to EGFR inhibitors^[72]^.

After transformation to SCLC, the median OS was 9-10 months with significant lower OS in the non–EGFR-mutant group. Once the tumor is transformed its survival and response to treatment seems comparable to that of classical SCLC. For example, in 39 patients the median time from diagnosis of NSCLC to the transformation to SCLC was 19 months and the median survival after SCLC diagnosis 6 months^[73]^. Thus, atypical SCLC features are visible after transformation and a lower response rate to etoposide/carboplatin EC treatment than classical SCLC is detected. At our institution we have established two cell lines from NSCLC patients progressing after osimertinib TKI treatment which have transformed to SCLC as proved by detection of the typical markers. Single transformed SCLC cells of these lines proved to be highly chemosensitive to cisplatin and topotecan but in tissue culture large spheroids similar to the SCLC CTC tumorospheres were detected and may be held responsible for chemoresistance and a dismal prognosis after a NSCLC-SCLC switch [Figure 1B].

## Spheroids as hypoxic niche

Tumor cells may reside in a protected environment and recur after prolonged times upon reactivation. For tumor like breast cancer this niche is located at less vascularized and partially hypoxic regions within the bone marrow^[74]^. This tumor dormancy is classically defined as the arrest of tumor growth in the primary site or in metastatic dissemination. In cellular dormancy, cancer cells are in a quiescent state characterized by minimum proliferation, minimum death and reversibility. Dormant cancer cells are likely resistant to conventional therapies which target actively proliferating cells. This may be a property common to all cell lines since cancer tissue-originated spheroids could be reversibly held dormant for at least 7 days in hypoxia without growth factor stimulation^[75]^. Under hypoxic conditions, tumor cells adapt a more aggressive tumor phenotype including the activation of DNA damage repair proteins, altered metabolism, and decreased proliferation^[76]^.

Tumorospheres seem to fulfil the preconditions for a protected niche for dormant tumor cells: hypoxic conditions, quiescent cells and a protective cover by outer layers which exhibits continuous shedding of tumor cells and fragments (unpublished observation). Thus, in SCLC cells may not seek protection as single cells in the bone marrow but seem to be able to form a hypoxic niche themselves that is not amenable to eradication by chemotherapy^[77]^.

## Relation of SCLC CTCs to cancer stem cells and epithelial-mesenchymal trnsition phenotypes

Spheroids are small multicellular aggregates formed *in vitro* by cells from most cancers. Diverse methods have been used to demonstrate cancer small cell clusters/spheroids consisting of up to 10 cells in the blood of metastatic cancer patients but filtration/image analyses of unprocessed blood showed a size range of up to 30-60 µm obviously assembled by a larger number of tumor cells^[78]^. SCLC CTC tumorospheres reach sizes exceeding 500-1000 µm and, *in vivo*, may be the product of the smaller spheroids trapped in the microvasculature. Spheroid-forming potential has been ascribed to cancer stem cells (CSCs) as reported for neurospheres, mammospheres and structures observed in other cancers^[79]^. The possibility that the spheroids enclose and protect (CSCs) expressing the characteristic SOX2, NANOG and OCT4 markers have bee raised but in the case of the SCLC CTC cell lines no CSC features were found^[55]^.

Besides SCLC, the other cancer that is distinguished by extremely high numbers of CTCs is the highly aggressive IBC which can metastasize via clusters^[80]^. In this case, the formation of clusters has been linked to a hybrid epithelial/mesenchymal (E/M) phenotype of cells. In IBC, CTC clusters are launched as aggregates of up to 20 cells into the bloodstream which are able to traverse capillary constrictions as single-file chains^[81]^. Cells in a hybrid E/M phenotype retain at least some levels of E-cadherin and co-express epithelial and mesenchymal markers. However, at least two of the SCLC CTC cell lines exhibit high expression of cadherin and low expression of vimentin constituting a transition epithelial-mesenchymal transition (EMT) phenotype at most that ultimately is not compatible with formation of the tumorospheres^[55]^. EMT has been found to be dispensable for metastasis in experimental animal models and, so far, clinical attempts to target CSCs were not effective to prolong survival^[82,83]^.

## Conclusion

SCLC CTC spheroids seem not to rely on features of EMT or CSCs phenotypes for drug resistance. The circulating SCLC clusters/small spheroids may grow to large tumorospheres after entrapment in capillaries and start the process of extravasation with help of proteolytic enzymes. In addition to the overexpression of matrix metalloproteinase-9, SCLC CTC lines release cathepsin S which has been involved in brain metastasis which is frequently observed for this tumor type^[84]^. Assembly of SCLC cells into tumorospheres seem to be sufficient for broad-range chemoresistance and, unfortunately, the clinical strategies to attack and eliminate such aggregates *in vivo* are not available yet. Patient-derived xenograft models employing CTCs should assist in the development of new therapeutic modalities directed to spheroids^[85]^.

## Declarations

### Copyright

© The Author(s) 2019.
